# Relations among Stigma, Quality of Life, Resilience, and Life Satisfaction in Individuals with Burn Injuries

**DOI:** 10.3390/ebj3010012

**Published:** 2022-02-15

**Authors:** Jack D. Watson, Paul B. Perrin

**Affiliations:** 1 Department of Psychology, Virginia Commonwealth University, Richmond, VA 23284, USA; watsonjd4@vcu.edu; 2 Department of Physical Medicine and Rehabilitation, Virginia Commonwealth University, Richmond, VA 23284, USA

**Keywords:** burn injury, stigma, resilience, life satisfaction, quality of life

## Abstract

This study examined the relations among burn stigma, quality of life, resilience, and life satisfaction, hypothesizing that higher stigma and lower burn-related quality of life would lead to lower life satisfaction; however, resilience would moderate this relation. A sample of 89 participants was recruited from an outpatient clinic of a burn center in a critical care hospital. Participants completed a battery of measures assessing these constructs. Results suggested that burn stigma was associated with reduced life satisfaction after accounting for other variables. Multiple regression models found that burn stigma predicted both affect and body image but not interpersonal relationship quality or sexuality. Interpersonal relationship quality, sexuality, affect, and body image all predicted life satisfaction. Both affect and body image partially accounted for the relation between stigma and life satisfaction, and resilience accounted for the relation between stigma and affect. Findings reinforce previous literature that has shown a relationship between stigma and life satisfaction but also emphasizes the role of resilience and burn-related quality of life. Individuals who experience a burn injury may have innate resilience abilities, which allow them to bounce back from stressors; thus, resilience can be a targeted strength to bootstrap in order to improve adjustment outcomes.

## 1. Introduction

Worldwide, non-fatal burn injuries are a leading cause of morbidity [1], with over 400,000 burns occurring in the US each year [1,2]. While the survival rate for burn injuries is nearly 97% [2], individuals with burn injuries often experience stigma related to their injury [3,4], negative psychosocial outcomes [5], dysfunctional coping [6], pain [7,8], and decreased physical functioning [5].

Stigma’s frequent and pervasive presence after burn injury is well-established. Stigma is defined as the “prejudice and discrimination that results from endorsing negative stereotypes about people” with a burn injury [9]. Families of individuals with burn injuries often reported feelings of fear, shame, and guilt which may manifest through various stigmatizing verbalizations and behaviors [10]. Individuals with burn injuries may be stared at or ridiculed [11]. In particular, individuals with facial/neck burns or amputations as a result of a burn injury are at increased risk for experiencing stigma [4]. While the effects of stigma for individuals with burn injury have not been well investigated, research has shown that stigma may lead to worse psychological well-being [12]. Research on the effects of stigma for various other forms of injury or disability is also telling, linking stigma to reduced life satisfaction [13], reduced daily activity [14], and lack of belonging [15].

Individuals who sustain a burn may face a number of physical and psychosocial difficulties that reduce their quality of life. Pain and scarring have become the main foci of burn wound care as they can lead to a host of other complications, including heat intolerance, reduced range of motion, and disfigurement, all of which can reduce a burn survivor’s quality of life. Sexual dysfunction is also a common concern and can negatively impact romantic relationships and self-esteem [16]. These sequelae are often compounded by negative body image [17] and difficulty with social interactions, which can lead to feelings of isolation and loneliness [11]. All of these difficulties, without proper support and coping strategies, can lead to poor psychosocial adjustment and mental health [6]. While quality of life is an important predictor of both life satisfaction and psychological health for individuals with burn injuries, to our knowledge, no study to date has examined the mediating role of quality of life in the relations among stigma, resilience, and life satisfaction.

Individuals with burn injury face a host of challenges, but most adapt positively despite these changes [18]. Many describe the initial hospitalization experience as trying to endure the pain of the burn, while coping post-discharge is often characterized by finding ways to deal with the various consequences associated with the injury (e.g., scarring, disfigurement, heat sensitivity, pain, etc.) [18]. While posttraumatic stress disorder (PTSD) can be present at discharge, rates are often higher at follow-up, indicating a need for greater psychosocial support following discharge [18,19]. Research has shown that psychosocial adjustment for individuals with burn injuries was strongly associated with personality characteristics, body image, and self-esteem [18,19] and that resilience may be an especially important factor in facilitating positive coping [20].

Resilience is a broadly defined but underutilized construct within the burn literature [21]. In fact, a 2016 review by Kornhaber and colleagues found only 10 studies examining resilience in the context of burn injury, and one of the studies used data collected from caregivers rather than the individual with burn directly [21]. Resilience, or the ability to cope with stress [22], has been shown to be a protective factor for individuals with burn injuries, with research linking resilience to positive subjective well-being [23], increased self-esteem [24], better social relations, and positive coping strategies [21]. While the relation between resilience and stigma has been examined within a number of populations with diverse health conditions, very little research has examined this relation within the context of burn injuries [21]. In fact, to the authors’ knowledge, this is only the second study to investigate the relation between stigma and resilience among individuals with burn injuries, while the other was specifically focused on women with facial burns [20]. Habib and colleagues showed a positive relation between resilience and coping with burns as well as social comfort, whereas stigma was negatively related to social comfort, coping, and resilience [20].

Previous literature has linked the experience of stigma to reduced life satisfaction in individuals with mental illness [13]. Corry and colleagues [17] note that this relation appears to be at work for individuals with burn injuries and called for greater investigation of this phenomenon. A rich body of literature on the stigma of mental illness demonstrates that the experience of stigma is strongly associated with a decrease in quality of life [25] and that stigma predicts negative quality of life longitudinally [26]. Similarly, research has shown lower health-related quality of life to be associated with lower life satisfaction for individuals with disabilities more generally [27], and resilience has been connected to subjective well-being, higher self-esteem, and increased social connectedness for individuals with burn injuries [23,24]. A review by Kornhaber et al. [21] highlighted the importance and possible impacts of resilience in individuals with burn injuries and called for greater investigation into the construct. Thus, the purpose of the current study was to examine the relations among these constructs for individuals with burn injuries. It was specifically hypothesized prior to analyses that greater experiences with stigma would lead to reduced burn-related quality of life across multiple domains, which in turn would lead to lower life satisfaction. It was further hypothesized that different aspects of burn-related quality of life would mediate the effect of stigma on life satisfaction and that resilience would moderate these mediations (i.e., conditional indirect effects).

## 2. Materials and Methods

### 2.1. Participants

This study was approved by the sponsoring university’s institutional review board. Participants were recruited from an outpatient clinic of a verified burn center in a critical care hospital situated in a major university. Eligibility requirements were: (a) 18 years of age or older and (b) currently a patient in the outpatient burn clinic (i.e., have sustained a burn requiring treatment). There were no exclusion criteria based on the mechanism or type of burn injury, time since burn injury, or psychiatric conditions. All participants appeared able to speak English when interacting with data collectors. The sample included 89 participants, with their characteristics presented in Table 1.

### 2.2. Measures

Internalized Stigma of Burn Injury Brief Version (ISBI-9) [9]. The ISBI-9 is a brief, modified version of the ISMI-9 originally created to assess the stigma associated with mental illness. While the ISBI-9 has not been psychometrically evaluated, the ISMI-9 has demonstrated good psychometric properties and construct validity for generalized internalized stigma [9]. The current study modified the items to measure burn stigma (substituting “burn injury” for “mental illness”). Items are rated on a 4-point Likert-type scale with higher scores indicating greater burn stigma. Example items include: “I feel out of place in the world because I have a burn injury” and “Negative stereotypes about people with burn injury keep me isolated from the ‘normal’ world.” Reliability for this sample was good, with a Cronbach’s alpha of 0.86.

Burn Specific Health Scale-Brief (BSHS-B) [28]. The BSHS-B is a 40-item version of the BSHS [29] used to assess various aspects of burn-related quality of life for individuals with burn injuries. The BSHS is considered a valid and reliable measure of quality of life for individuals with burns [29], and the BSHS-B has been shown to have similar validity [28]. The BSHS-B has 9 subscales, but only 4 were used for analyses in this study: 7-item Affect (“I often feel sad or blue”), 4-item Interpersonal Relationships, (“My family would be better off without me”), 3-item Sexuality (“I am simply not interested in sex anymore”), and 4-item Body Image (“My general appearance really bothers me”). Items were scored on a 5-point Likert-type scale ranging from 0 (extremely) to 4 (not at all) and summed with lower scores indicating worse outcomes. Cronbach’s alphas ranged from 0.68 to 0.85; however, this should be interpreted with caution as the lowest was from the 3-item subscale.

Satisfaction with Life Scale (SWLS) [30]. The SWLS is a 5-item scale used to assess global satisfaction with life. The SWLS has demonstrated acceptable psychometric properties and is highly associated with other measures of subjective well-being [30]. Items are scored ranging from 1 (Strongly Disagree) to 7 (Strongly Agree) and summed with higher scores indicating greater satisfaction with life. The scale demonstrated good internal consistency, with a Cronbach’s alpha of 0.88.

Brief Resilience Scale (BRS) [22]. The BRS is a 6-item measure of an individual’s ability to bounce back from stress. The BRS has demonstrated sound psychometric properties and the ability to distinguish between individuals with high and low resilience [22]. The item response options range from 1 (Strongly disagree) to 5 (Strongly agree). Items are summed with higher scores indicating greater resilience in the face of stress. The scale demonstrated good internal consistency, with a Cronbach’s alpha of 0.81.

### 2.3. Procedure

Institutional review board approval was received. Recruitment occurred between July 2013–February 2014. Research assistants (RAs) were on site at the outpatient burn clinic during normal clinic hours and approached potential participants while they waited for their outpatient appointment. RAs explained the study, screened for eligibility, and obtained written informed consent after participants had read an informed consent form outlining the purpose of the study (“to learn about and document the process of adjusting to burn injury”), a description of the study and their involvement, potential risks and discomfort, confidentiality, voluntary participation and withdrawal, and potential benefits to the burn community of their responses and the resulting research. After consent was obtained, participants were given a 30-min pen-and-paper questionnaire that they completed on their own and did not take into the appointment. Each measure contained general questions without instructions for participants to contemplate a specific time period. As such, participants were free to interpret the questions as pertaining globally to their life.

### 2.4. Data Analyses

**Figure 1:**
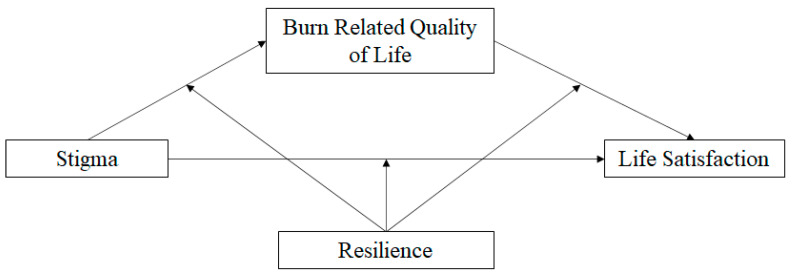
Moderated Mediation Model.

IBM SPSS 27 was used for all statistical analyses. Four mediational models (Figure 1) were run using the Hayes PROCESS macro Model 4 [31] with 5000 bootstrap samples to examine the relations among stigma, burn-related quality of life (with interpersonal relationships, sexuality, affect, and body image as the four mediators), and life satisfaction in individuals with burn injuries. For significant mediations only, the Hayes PROCESS macro Model 59 [31] was used to investigate the possible moderating effect of resilience on these mediations (Figure 1). Where moderated mediations were present, the Hayes PROCESS macro Model 1 [31] was used to determine where in the overall mediational model the moderating effect occurred.

## 3. Results

### 3.1. Mediations

#### 3.1.1. Interpersonal Relationships

The first mediation examined whether quality of interpersonal relationships mediated the relation between stigma and life satisfaction. Stigma (b = −0.07, *p* = 0.165) did not significantly predict interpersonal relationships, but interpersonal relationships (b = 1.40, *p* < 0.001) predicted life satisfaction. Stigma (b = −0.58, *p* < 0.001) also predicted life satisfaction even after accounting for interpersonal relationships. The overall model was significant, F(2, 86) = 22.52, *p* < 0.001, R^2^ = 0.34. Approximately 34% of the variance in life satisfaction was accounted for by stigma and interpersonal relationships. The 95% confidence interval (CI) for the indirect effect included 0 (b = −0.10, 95% CI = [−0.26, 0.02]), suggesting that quality of interpersonal relationships did not mediate the effect of stigma on life satisfaction.

#### 3.1.2. Sexuality

The second mediation examined whether sexuality mediated the relation between stigma and life satisfaction. Stigma (*b* = −0.07, *p* = 0.191) did not significantly predict sexuality, but sexuality (*b* = 0.74, *p* = 0.017) predicted life satisfaction. Stigma (*b* = −0.63, *p* < 0.001) also predicted life satisfaction even after accounting for sexuality. The overall model was significant, *F*(2, 86) = 18.29, *p* < 0.001, *R*^2^ = 0.17. Approximately 17% of the variance in life satisfaction was accounted for by stigma and sexuality. The 95% CI for the indirect effect included 0 (*b* = −0.05, 95% CI = [−0.16, 0.02]), suggesting that sexuality did not mediate the effect of stigma on life satisfaction.

#### 3.1.3. Affect

The third mediation examined whether affect mediated the relation between stigma and life satisfaction. Stigma (*b* = −0.45, *p* < 0.001) predicted affect, and affect (*b* = 0.65, *p* < 0.001) predicted life satisfaction. Additionally, stigma (*b* = −0.40, *p* = 0.014) still predicted life satisfaction even after accounting for affect. The overall model was significant, *F*(2, 86) = 21.38, *p* < 0.001, *R*^2^ = 0.33. Approximately 33% of the variance in life satisfaction was accounted for by stigma and affect. The 95% CI for the indirect effect did not include 0 (*b* = −0.29, 95% CI = [−0.56, −0.11]), suggesting that affect partially mediated the effect of stigma on life satisfaction.

#### 3.1.4. Body Image

The fourth mediation examined whether body image mediated the relation between stigma and life satisfaction. Stigma (*b* = −0.21, *p* = 0.010) predicted body image, and body image predicted life satisfaction (*b* = 0.76, *p* < 0.001). Additionally, stigma (*b* = −0.52, *p* = 0.001) predicted life satisfaction even after accounting for body image. The overall model was significant, *F*(2, 86) = 17.91, *p* < 0.001, *R*^2^ = 0.29. Approximately 29% of the variance in life satisfaction was accounted for by stigma and body image. The 95% CI for the indirect effect did not include 0 (*b* = −0.16, 95% CI = [−0.37, −0.04]); suggesting that body image partially mediated the effect of stigma on life satisfaction. All mediation results are reported in Table 2.

### 3.2. Moderated Mediations

#### 3.2.1. Affect

The first moderated mediation model assessed the addition of resilience as a moderator of the relations among stigma, affect, and life satisfaction. The overall model was significant, *F*(5, 83) = 9.88, *p* < 0.001, *R*^2^ = 0.37. There was a significant indirect effect of stigma on life satisfaction through affect at low levels of resilience; however, at high levels of resilience, there was not, reflecting a moderated mediation or conditional indirect effect (Table 3). Because of this moderated mediation, we ran 3 follow-up moderations to determine where in the model the moderation might be occurring.

**Figure 2:**
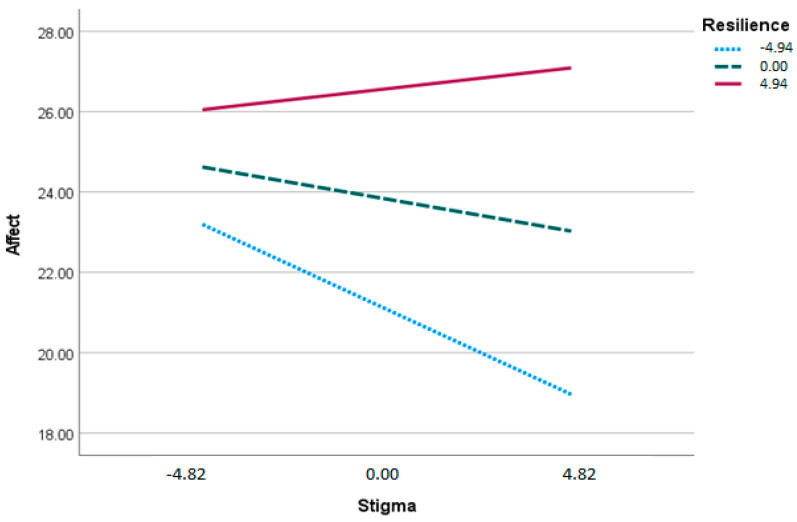
Moderated mediation. Although in the actual analyses, resilience was left continuous (with the exception of Model 59), for graphing purposes only, variables were mean-centered and groups created by resilience level at ±1 standard deviation.

The first moderation examined stigma to affect moderated by resilience. The overall model was significant, *F*(3, 85) = 20.92, *p* < 0.001, *R*^2^ = 0.42. The interaction term was also significant (*b* = 0.06, *p* = 0.009), indicating that resilience moderates the relation between stigma and affect (Figure 2). The second moderation examined stigma to life satisfaction moderated by resilience. The overall model was significant, *F*(3, 85) = 12.60, *p* < 0.001, *R*^2^ = 0.31. The interaction term was not significant (*b* = 0.02, *p* = 0.550), indicating that resilience did not moderate the relation between stigma and life satisfaction. The final moderation examined affect to life satisfaction moderated by resilience. The overall model was significant, *F*(3, 85) = 14.86, *p* < 0.001, *R*^2^ = 0.34. The interaction term was not significant (*b* = −0.02, *p* = 0.562), indicating resilience did not moderate the relation between affect and life satisfaction.

#### 3.2.2. Body Image

The second model assessed the addition of resilience as a moderator in the relations among stigma, body image, and life satisfaction. The overall model was significant, *F*(3, 83) = 9.88, *p* < 0.001, *R*^2^ = 0.37. There was a significant indirect effect of stigma on life satisfaction through body image across all levels of resilience, indicating the absence of a moderated mediation (Table 3).

## 4. Discussion

The current study examined the relations among stigma, burn-related quality of life (quality of interpersonal relationships, sexual interest, affect, and body image), and life satisfaction; then, for the two significant mediational models (affect and body image), we examined resilience as a moderator of these relations. Although stigma did not predict the quality of interpersonal relationships or sexual interest, it did predict both affect and body image. Furthermore, all four aspects of burn-related quality of life predicted life satisfaction. Both affect and body image partially mediated the relation between stigma and life satisfaction. Resilience moderated the relation between stigma and affect, reducing the negative effect of stigma on life satisfaction through affect at high levels of resilience. Thus, the model theoretically demonstrates the following sequence of events: (1) an individual with a burn experiences stigma, (2) there is a resulting negative impact on the individual’s body image or affect, (3) this reduces life satisfaction, and (4) for affect only, resilience buffers the relation between stigma and affect.

To our knowledge, this is only the second study to examine the relation between stigma and resilience in individuals with burn injuries and the first to do so within the context of life satisfaction and quality of life in a sample of both men and women [20,21]. This study builds on the previous limited resilience literature within the context of burn injuries and assists in answering the call from previous researchers to continue to elucidate the important role of resilience in the lives of individuals with burn injury [20,21]. Furthermore, this study is also the first to examine the mediating role of quality of life in the relation between stigma and life satisfaction for individuals with burn injuries.

Stigma [3,4,12], reduced body image [17], poor psychosocial outcomes [12], and dysfunctional coping [6] are all major concerns facing individuals with burn injuries. The current study’s findings supported previous research linking higher levels of stigma and lower levels of quality of life to lower life satisfaction [13,27]. Both sexuality and interpersonal relationships predicted life satisfaction, in line with previous research [11,16]. Previous research has shown that changes in appearance following burn injury and worse mental health are closely related to stigma [11,12], and while the current study reinforced these findings, it also demonstrated the mediational role of both affect and body image in the relation between stigma and life satisfaction. This helps explain some of the cascading influence of stigma as it may act through both negative affect and body image and indicates that both reduced body image and negative affect are likely to be found in the presence of stigma. Additionally, resilience has been shown to be a protective factor for individuals with burn injuries [21,23,24], a finding reinforced in the current study through the moderating effect of resilience on affect’s mediation in the relation between stigma and life satisfaction. This series of findings indicate several areas of possible intervention for individuals with burn injury.

### 4.1. Implications

This study successfully illuminated the relations among burn stigma, quality of life, resilience, and life satisfaction. Individuals with burn injuries may experience stigma [3,4,12] and reduced quality of life [6], which can lead to a reduction in life satisfaction, as well as other psychosocial complications. One way to address the decrease in life satisfaction might be to assist in reducing stigma. Burn clinics may wish to institute routine culturally and disability-sensitive training for healthcare providers to help reduce stigma or belittling language encounters in the clinical environment. They may also incorporate interventions that help diminish invalidating or stigmatizing verbalizations and behaviors from the individual’s family or caregivers. This may be especially relevant for caregiver training in which potentially stigmatizing behaviors (like trying to hide the burn due to fear or shame) might be addressed and for couples’ therapy to help facilitate understanding from those who have not experienced a burn. Additionally, since both interpersonal relationship quality and sexual interest were significant predictors of life satisfaction, this indicates another target area for intervention within the context of couples and partner-caregivers. Greater attention to the quality of relationship and sexuality may help increase general satisfaction with life for individuals with burn injuries.

Both affect and body image partially mediated the relation between stigma and life satisfaction, again indicating areas for targeted intervention. Focusing therapy on positive coping skills, self-acceptance, and strength-based approaches to recovery may help attenuate this relation and lead to increased life satisfaction. Resilience is a broad construct, particularly within the burn literature [21], and may encompass a number of constructs, including social support, acceptance, positive outlook, social comfort, identity, grit, and feelings of belonging. Thus, individuals with burn injuries may be able to face or overcome many challenges due to their social network, personal identity, disability identity, and innate characteristics like grit, positivity, and determination. These resilience categories all offer unique ways of coping with stress. Increasing a patient’s resilience in the face of stigma might also be helpful, particularly as it pertains to decreasing the effects of negative affect. Identity-based group identification, such as having a positive disability identity, can be a significant protective factor with a number of benefits, including reduced effects of stigma [32]. It is therefore recommended that burn clinics help facilitate community integration and social comfort for individuals with burn injuries. As such, scaffolding any aspect of resilience may assist individuals with burn injuries in developing healthy coping mechanisms and can be accomplished by any member of the healthcare team. Thus, it is recommended that treatments facilities (acute, rehabilitation, outpatient, etc.) institute programs, like those listed above, to help support individuals with burn injuries.

### 4.2. Limitations and Future Directions

The current study used only cross-sectional data with 89 participants. A longitudinal study would better assess the effects of stigma and burn-related quality of life on life satisfaction as well as the possible moderating role of resilience over time; such a study would also help more strongly infer causality in the relations found among the current variables. Due to the cross-sectional nature of the present study, we were unable to rule out a possible reverse ordering of the variables of interest. That is, it is possible that stigma mediates the relation between quality of life and life satisfaction. As a result, caution should be applied in interpreting causal effects within the relations found in the current study. Furthermore, psychosocial adjustment is malleable, particularly during the transition from rehabilitation to home [18,19]. As such, a longitudinal study might help uncover possible fluctuations in adjustment, coping styles, and resilience that the present study was unable to capture. The current sample was largely male (71%) and White (71%), limiting the study’s generalizability. A relatively small percentage had facial burns (27%), and since facial burns are a particular risk factor for stigma [4], a similar study conducted only with individuals with facial (or highly visible) burns may yield different results. It is noteworthy that eight participants refused or were unable to describe the location of their injury. Although this may be due to the burn occurring on a stigmatized part of their body or that the burn was too complex to be described in a straightforward manner, the ultimate source of this missingness is unknown. Because the data were collected anonymously, unfortunately, medical records could not be accessed in order to abstract this information. Future research may wish to incorporate a larger, more diverse sample or target a sample consisting of those most at risk to experience stigma related to burns.

## 5. Conclusions

This was the first study to examine the moderating role of resilience on the relations among stigma, burn-related quality of life, and life satisfaction in individuals with burn injuries. While the current study findings reinforce previous literature that has shown a relation between stigma and life satisfaction, the current findings help illuminate the role of resilience and burn-related quality of life in these relations. Specifically, this study highlights that individuals who experience a burn injury may have innate abilities that allow them to bounce back from stressors and that this resilience can be a targeted strength to help improve outcomes. It is recommended that rehabilitation clinicians use these results to guide clinical practice by implementing integration-focused, positive, and affirming interventions for individuals with burn injuries.

## Figures and Tables

**Table 1:** Sample Characteristics.

Characteristics	(*N* = 89)
Age, *M* (*SD*)	41.20 (14.45)
Sex, n (%)	
Male	63 (70.80)
Female	26 (29.20)
Race, n (%)	
White	63 (70.80)
Black	21 (23.60)
Asian/Asian-American	2 (2.20)
Hispanic/Latino	1 (1.10)
Multiracial/Multiethnic	1 (1.10)
Unknown/Refused	1 (1.10)
Education, n (%)	
Grade School	3 (3.40)
High School Graduate	32 (36.00)
Some College AA/AS/Technical	26 (29.20)
College Graduate	19 (21.30)
Master’s Degree	5 (5.60)
Doctorate	3 (3.40)
Unknown/Refused	1 (1.10)
Injury Location, n (%)	
Hands Only	29 (32.60)
Not on Face or Hands	28 (31.50)
Both Face and Hands	19 (21.30)
Face Only	5 (5.60)
Unknown/Refused	8 (9.0)
Months Since Injury, *M* (*SD*)	4.70 (7.14)
Range in months	1–30
Participants at 1-month post-injury, n (%)	52 (59.1)
Participants within 7 months post-injury, n (%)	71 (80.7)
Total Body Surface Area Burned, n (%)	
0–10%	52 (58.4)
11–20%	14 (15.7)
21–30%	12 (13.5)
31–40%	4 (4.5)
41–50%	1 (1.1)
51–60%	1 (1.1)
61–70%	1 (1.1)
<70%	1 (1.1)
Unknown/Refused	3 (3.4)
Employed Prior to Injury, n (%)	
Full-time	57 (64.0)
Unemployed	20 (22.5)
Part-time	6 (6.70)
Student	3 (3.40)
Unknown/Refused	3 (3.40)
Employed at Time of Research, n (%)	
Full-time	44 (49.40)
Unemployed	28 (31.50)
Part-time	4 (4.50)
Student	4 (4.50)
Unknown/Refused	9 (10.10)

**Table 2:** Mediational Models.

Stigma to Life Satisfaction Through Burn Related Quality of Life				
Interpersonal Mediation				
	*b*	*p*	LCI	UCI
Stigma to Interpersonal	−0.07	0.165	−0.18	0.03
Interpersonal to Life Satisfaction	1.40	<0.001	0.81	1.99
Stigma to Life Satisfaction (after Interpersonal)	−0.58	<0.001	−0.87	−0.29
Direct Effect of Stigma	−0.68	<0.001	−1.00	−0.37
Indirect Effect of Stigma	−0.10		−0.26	0.02
Sexuality Mediation				
Stigma to Sexuality	−0.07	0.191	−0.18	0.04
Sexuality to Life Satisfaction	0.74	0.017	0.14	1.35
Stigma to Life Satisfaction (after Sexuality)	−0.63	<0.001	−0.94	−0.32
Direct Effect of Stigma	−0.68	<0.001	−1.00	−0.37
Indirect Effect of Stigma	−0.05		−0.16	0.02
Affect Mediation				
Stigma to Affect	−0.45	<0.001	−0.66	−0.23
Affect to Life Satisfaction	0.65	<0.001	0.36	0.93
Stigma to Life Satisfaction (after Affect)	−0.40	0.014	−0.71	−0.08
Direct Effect of Stigma	−0.68	<0.001	−1.00	−0.37
Indirect Effect of Stigma	−0.29		−0.56	−0.11
Body Image Mediation				
Stigma to Body Image	−0.21	0.010	−0.37	−0.05
Body Image to Life Satisfaction	0.76	<0.001	0.36	1.15
Stigma to Life Satisfaction (after Body Image)	−0.52	0.001	−0.83	−0.21
Direct Effect of Stigma	−0.68	<0.001	−1.00	−0.37
Indirect Effect of Stigma	−0.16		−0.37	−0.04

Note. *b* = beta; LCI = lower confidence interval; UCI = upper confidence interval.

**Table 3:** Conditional Indirect Effects of Stigma on Life Satisfaction through Affect and Body Image at Levels of Resilience (±1 Standard Deviation).

Resilience	Estimate	95% CI
Affect		
18.00	−0.21	−0.45 to −0.05
23.00	−0.05	−0.20 to 0.01
27.00	0.03	−0.10 to 0.17
Body Image		
18.00	−0.02	−0.17 to 0.09
23.00	−0.02	−0.18 to 0.05
27.00	−0.02	−0.30 to 0.07

## Data Availability

Data are available from the corresponding author upon request.
